# Identification of new reference genes with stable expression patterns for gene expression studies using human cancer and normal cell lines

**DOI:** 10.1038/s41598-021-98869-x

**Published:** 2021-09-30

**Authors:** Gergely Attila Rácz, Nikolett Nagy, József Tóvári, Ágota Apáti, Beáta G. Vértessy

**Affiliations:** 1 Department of Applied Biotechnology and Food Sciences, Budapest University of Technology and Economics, Budapest, Hungary; 2 Institute of Enzymology, Research Center for Natural Sciences, Eötvös Loránd Research Network, Budapest, Hungary; 3 Department of Biochemistry, Institute of Biology, Faculty of Science, Eötvös Loránd University, Budapest, Hungary; 4 Department of Experimental Pharmacology, National Institute of Oncology, Budapest, Hungary

**Keywords:** Gene expression analysis, Reverse transcription polymerase chain reaction, Cell culture, Cancer models

## Abstract

Reverse transcription—quantitative real-time PCR (RT-qPCR) is a ubiquitously used method in biological research, however, finding appropriate reference genes for normalization is challenging. We aimed to identify genes characterized with low expression variability among human cancer and normal cell lines. For this purpose, we investigated the expression of 12 candidate reference genes in 13 widely used human cancer cell lines (HeLa, MCF-7, A-549, K-562, HL-60(TB), HT-29, MDA-MB-231, HCT 116, U-937, SH-SY5Y, U-251MG, MOLT-4 and RPMI-8226) and, in addition, 7 normal cell lines (HEK293, MRC-5, HUVEC/TERT2, HMEC, HFF-1, HUES 9, XCL-1). In our set of genes, we included SNW1 and CNOT4 as novel candidate reference genes based on the RNA HPA cell line gene data from The Human Protein Atlas. HNRNPL and PCBP1 were also included along with the „classical” reference genes ACTB, GAPDH, IPO8, PPIA, PUM1, RPL30, TBP and UBC. Results were evaluated using GeNorm, NormFiner, BestKeeper and the Comparative ΔCt methods. In conclusion, we propose IPO8, PUM1, HNRNPL, SNW1 and CNOT4 as stable reference genes for comparing gene expression between different cell lines. CNOT4 was also the most stable gene upon serum starvation.

## Introduction

Quantitative polymerase chain reaction (qPCR) is often the method of choice when quantifying individual nucleic acids due to its high sensitivity, excellent specificity and good reproducibility^1–5^. For gene expression studies, qPCR is coupled with reverse transcription (RT) for the conversion of RNA to DNA, which can be applied to the qPCR reaction^6,7^. While being an appropriate and reliable method, RT-qPCR still requires thorough optimization and validation steps^8,9^. The MIQE guidelines were published more than a decade ago with the intention to aid researchers in the process of designing, performing and interpreting qPCR experiments and with the aim of ensuring reliability of results and increasing experimental transparency^10,11^.

To gain reliable results, a proper normalization method needs to be used. Normalizing to the amount of starting material (e.g. cell count, tissue mass) is possible, however, it implies that inherent variation in the yield of RNA extraction as well as in the efficiency of RT and qPCR still needs to be accounted for^12–14^. Moreover, the cellular mRNA pool and total RNA pool also show variations under different experimental conditions^2,3,15,16^. To overcome these issues, normalization to internal control genes or reference genes is performed in the vast majority of studies. Ideally, a housekeeping gene is selected for normalization whose expression is invariant. Unfortunately, no ideal housekeeping genes were identified, since the expression of all genes investigated was shown to be dependent on the origin and type of cells or tissues, developmental stages and experimental conditions^15,17–22^. To minimize experimental bias, two or more reference genes have to be selected that have minimal variability in all conditions included in the study^10,23,24^. The suitability of reference genes thus has to be verified for each RT-qPCR experiment^10,25^.

The cytoskeletal protein actin (beta isoform) (ACTB) and the glycolytic enzyme glyceraldehyde-3-phosphate dehydrogenase (GAPDH) genes are among the most widely used reference genes in gene expression studies. However, numerous studies indicated that the expression profiles of these two genes and also expression of other „classical” reference genes show considerable variations^3,26–34^. Finding proper reference genes for comparing gene expression in different cell lines, primary cell cultures or tissues is especially challenging due to high biological variability^25^. To address this problem, a systematic approach is adequate^14,21,22,34–37^. In CHO cell lines Brown et al. selected five candidate reference genes based on transcriptomic datasets, and studied the expression stability of these genes and three other widely used reference genes in 20 different commonly applied experimental conditions and suggested novel reference genes for experiments using CHO cell lines^38^. Jo et al. investigated large-scale expression data in The Cancer Genome Atlas (TCGA) database—which contains 9,364 cancerous and 664 normal samples from 32 different cancer types—to identify novel reference genes with the most stable expression^34^. They concluded that most commonly used reference genes are not stable enough and suggested novel reference genes for cancer cell studies.

Here, our aim was to identify reference genes for both normal and cancer cell studies. Towards this aim, we analyzed the RNA HPA cell line gene data as part of The Human Protein Atlas^39,40^ to select genes with the lowest expression variation among 69 cell lines. The two top-ranking genes were included in our set of 12 candidate reference genes along with two genes recommended by Jo et al. for cancer research and eight „classical” reference genes. The suitability of reference genes was assessed for quantifying gene expression across a set of popular cell lines from diverse cancer types as well as finite and immortalized normal cell lines. GeNorm^24^, NormFinder^41^, BestKeeper^42^ and the Comparative ΔCt method^43^ were used to evaluate our results. In addition, we also examined the effect of serum starvation—as a commonly used experimental condition—on the expression of our candidate genes. We propose to include the CNOT4 and SNW1 genes in the reference gene panels for gene expression studies.

## Results

### Cell lines used in this study

Our aim was to select human cancer cell lines widely used in different studies in the literature. For this purpose, the cell line panels NCI-60 with additional cell lines, JFCR39, KuDOS 95 and LL-100 were considered. Additionally, popular human cell lines HeLa and SH-SY5Y were also included. A literature search was conducted to determine the number of publications in which the name of the cell line or any synonymous name appears in the title or the abstract (Supplementary Dataset 1). Based on these criteria, 13 widely used human cancer cell lines were selected for this study. Furthermore, we investigated 7 normal human cell lines, as well. The cell line HEK293 was classified as normal based on its origin. All cell lines included in our study are summarized in Table 1.

**Table 1:** Cell lines used in this study.

Accession (RRID)	Cell line	Category	Disease	Cell type
CVCL_0023	A-549	Cancer cell line	Lung adenocarcinoma	Alveolar basal epithelial cell
CVCL_0291	HCT 116	Cancer cell line	Colon carcinoma	Intestinal epithelial cell
CVCL_0030	HeLa	Cancer cell line	Human papillomavirus-related endocervical adenocarcinoma	Epithelial cell
CVCL_A794	HL-60(TB)	Cancer cell line	Adult acute myeloid leukemia	Promyelocyte
CVCL_0320	HT-29	Cancer cell line	Colon adenocarcinoma	Intestinal epithelial cell
CVCL_0004	K-562	Cancer cell line	Chronic myelogenous leukemia, BCR-ABL1 positive	Highly undifferentiated myeloid cell
CVCL_0031	MCF-7	Cancer cell line	Invasive breast carcinoma	Mammary gland luminal A epithelial cell
CVCL_0062	MDA-MB-231	Cancer cell line	Breast adenocarcinoma	Mammary gland basal B epithelial cell
CVCL_0013	MOLT-4	Cancer cell line	Adult T acute lymphoblastic leukemia	Precursor T-cell
CVCL_0014	RPMI-8226	Cancer cell line	Plasma cell myeloma	B lymphocyte
CVCL_0019	SH-SY5Y	Cancer cell line	Neuroblastoma	Neuron (dopaminergic/adrenergic)
CVCL_0021	U-251MG	Cancer cell line	Astrocytoma	Astrocyte
CVCL_0007	U-937	Cancer cell line	Adult acute monocytic leukemia	Monocyte
CVCL_0045	HEK293	Transformed cell line	Normal—transformed with Ad5	Adrenal precursor cell
CVCL_3285	HFF-1	Finite cell line	Normal	Foreskin fibroblast
CVCL_UW69	HMEC	Telomerase immortalized cell line	Normal—immortalized with TERT	Mammary epithelial cell
CVCL_0057	HUES 9	Embryonic stem cell	Normal	Embryonic stem cell from blastocyst
CVCL_9Q53	HUVEC/TERT2	Telomerase immortalized cell line	Normal—immortalized with TERT	Umbilical vascular endothelial cell
CVCL_0440	MRC-5	Finite cell line	Normal	Embryo lung fibroblast
CVCL_WM82	XCL-1	Induced pluripotent stem cell	Normal	Induced pluripotent stem cell

### Selection of reference genes and primers

Twelve reference genes were selected for our study. The RNA HPA cell line gene data from The Human Protein Atlas^39,40^ was analyzed to identify genes with relatively stable expression between 69 different cell lines as indicated by the coefficient of variation of normalized gene expression values (Supplementary Dataset 2). The most stable gene (SNW1) and the third most stable gene (CNOT4) according to our analysis were included in this study. The second most stable gene (Heterogeneous Nuclear Ribonucleoprotein K (HNRNPK)) was omitted because we decided to use another heterogeneous nuclear ribonucleoprotein gene (HNRNPL), already suggested as a proper reference gene^34^, to represent the set of HNRNP genes. For a proper ranking evaluation of candidate reference genes, it is important to avoid using genes under similar expression regulation as the methods assessing gene expression stability would preferentially select coregulated genes as the most stable ones. The PCBP1 gene was also included in our set as suggested^34^. Moreover, a list of the most commonly used reference genes was generated from which the ones with the lowest coefficient of variation (CV) value (cf “Methods”) were selected as candidate reference genes (Table 2).

**Table 2:** List of potential reference genes.

Gene symbol	Gene name	Ensembl gene ID	Function	CV	Rank	No. of articles
**SNW1**	**SNW domain-containing 1**	**ENSG00000100603**	**Signal transduction, regulation of transcription and splicing**	**0.189**	**1**	**0**
**CNOT4**	**CCR4-NOT transcription complex subunit 4**	**ENSG00000080802**	**Global transcriptional regulation, deadenylase, signal transduction, E3 ubiquitin ligase**	**0.205**	**3**	**0**
**PUM1**	**Pumilio RNA binding family member 1**	**ENSG00000134644**	**Regulation of the stability and function of specific mRNAs**	**0.235**	**8**	**11**
***PCBP1***	***Poly(rC) binding protein 1***	***ENSG00000169564***	***mRNA stabilization, alternative splicing, regulation of transcription and translation***	***0.291***	***161***	***2***
**IPO8**	**Importin 8**	**ENSG00000133704**	**Protein transport—nuclear import of proteins with a classical nuclear localization signal**	**0.336**	**637**	**13**
***HNRNPL***	***Heterogeneous nuclear ribonucleoprotein L***	***ENSG00000104824***	***mRNA splicing, stabilization, regulation of transcription and translation***	***0.347***	***791***	***1***
**TBP**	**TATA-box binding protein**	**ENSG00000112592**	**Transcription—general transcription factor**	**0.347**	**813**	**70**
**UBC**	**Ubiquitin C**	**ENSG00000150991**	**Protein catabolism—ubiquitylation of damaged/unfolded proteins**	**0.366**	**1150**	**29**
**PPIA**	**Peptidylprolyl isomerase A**	**ENSG00000196262**	**Protein folding—Cis–trans isomerization of proline imidic peptide bonds**	**0.402**	**1893**	**49**
**RPL30**	**Ribosomal protein L30**	**ENSG00000156482**	**Translation—component of the 60S ribosomal subunit**	**0.420**	**2294**	**4**
RPL13A	Ribosomal protein L13a	ENSG00000142541	Translation—component of the 60S ribosomal subunit	0.438	2715	35
YWHAZ	Tyrosine 3-monooxygenase/ tryptophan 5-monooxygenase activation protein zeta	ENSG00000164924	Signal transduction—central hub protein for many signal transduction pathways	0.462	3258	37
**ACTB**	**Actin beta**	**ENSG00000075624**	**Cytoskeletal structural protein**	**0.472**	**3484**	**113**
**GAPDH**	**Glyceraldehyde-3-phosphate dehydrogenase**	**ENSG00000111640**	**Metabolism—oxidoreductase in glycolysis and gluconeogenesis**	**0.492**	**3909**	**165**
PGK1	Phosphoglycerate kinase 1	ENSG00000102144	Metabolism—kinase in glycolysis and gluconeogenesis	0.497	4014	29
HMBS	Hydroxymethylbilane synthase	ENSG00000256269	Metabolism—heme biosynthesis	0.551	4966	22
HPRT1	Hypoxanthine phosphoribosyltransferase 1	ENSG00000165704	Metabolism—purine synthesis in salvage pathway	0.551	4973	65
EEF1A1	Eukaryotic translation elongation factor 1 alpha 1	ENSG00000156508	Translation—aminoacyl-trna delivery to the ribosome	0.557	5060	12
ALAS1	5'-aminolevulinate synthase 1	ENSG00000023330	Metabolism—heme biosynthesis	0.578	5397	10
SDHA	Succinate dehydrogenase complex flavoprotein subunit A	ENSG00000073578	Metabolism—part of the mitochondrial respiratory chain	0.590	5579	35
GUSB	Glucuronidase beta	ENSG00000169919	Metabolism—degradation of dermatan and keratan sulfates	0.713	7074	24
TFRC	Transferrin receptor	ENSG00000072274	Metabolism—cellular iron uptake	0.738	7316	10
B2M	Beta-2-microglobulin	ENSG00000166710	Immunity—β-chain of major Histocompatibility complex class I molecules	0.906	8802	78
POLR2A	RNA polymerase II subunit A	ENSG00000181222	Transcription—RNA polymerase	1.143	10,376	12

Coefficient of variation (CV) and the corresponding rank is calculated from the relative expression data in the RNA HPA cell line gene dataset from The Human Protein Atlas^39,40^. Genes are arranged in ascending order of the rank. The genes used in the present study are shown in bold. Genes suggested by Jo et al. are shown in *ita*l*ic*^34^. For details regarding the No. of articles, refer to “Methods”.

For the amplification of our candidate reference genes, three or four primer pairs were designed for each target. Whenever possible, primers were designed to be located on exon–exon junctions (intron-spanning primer design) or on two consecutive exons separated by an intron (intron-flanking primer design) to avoid amplification of genomic DNA contamination. For details of primer design, refer to “Methods”. Primer pairs were tested with temperature gradient qPCR, and the pairs with the lowest Cq values—that is, the primer pairs with maximal PCR efficiency—were selected for each target if specific PCR products were formed as indicated by melting curve analysis and agarose gel electrophoresis. Table 3 shows the relevant parameters of the primers designed for the selected references genes. The specificity of the PCR product was investigated initially with agarose gel electrophoresis (Supplementary Fig. S1), and checked routinely by performing melting curve analysis after amplification (Supplementary Fig. S2). Each PCR product appeared as a sharp band on agarose gel and was characterized with a single peak with melting curve analysis arguing for specific product formation.

**Table 3:** List of candidate reference genes and the corresponding primer parameters used in this study.

Gene symbol	Primer sequences (5'–3')	PCR product length (bp)	Tm of PCR products (°C)	Primer design	PCR amplification efficiency (%)	Regression Coefficient (R-squared)
SNW1	Fw: GCAGCTCCTGATAAGAGGTCG Rev: CCGAGGATTAGGAACACCGAG	87	78.0	Intron-spanning	95.8	0.9996
CNOT4	Fw: GTCCAAAACCTGACTGCATGTATC Rev: GGTGTTTACCCGCCTGCAT	87	80.8	Intron-spanning	96.3	0.9999
PUM1	Fw: TGCGGGAGATTGCTGGACAT Rev: GTGTGGCACGCTCCAGTTTC	87	80.4	Intron-flanking	98.4	0.9999
PCBP1	Fw: ATTCGCCGGAATTGACTCCA Rev: TGCCCAATAGCCTTTCACCT	49	86.4	Exonic	99.8	0.9998
IPO8	Fw: GGCATACAGTTTAACCTGCCAC Rev: CAGGAGAGGCATCATGTCTGTAA	118	78.6	Intron-spanning	92.5	0.9995
HNRNPL	Fw: CCAAGGCCTCTCTCAATGGG Rev: TTCAAGCGTGTAGGCTTTGC	82	80.0	Intron-spanning	97.9	0.9998
TBP	Fw: ATATAATCCCAAGCGGTTTGCTG Rev: AAAATCAGTGCCGTGGTTCG	66	79.8	Intron-spanning	97.4	0.9989
UBC	Fw: GGTCGCAGTTCTTGTTTGTGG Rev: TTCACGAAGATCTGCATTGTCAAG	60	78.4	Exonic	100.7	0.9998
PPIA	Fw: TGGGTTACTTCTGAAACATCACTTGT Rev: TTGACACTTCCTGGGACTGGA	85	75.1	Exonic	98.2	0.9999
RPL30	Fw: TTCTCGCTAACAACTGCCCA Rev: TGCCACTGTAGTGATGGACAC	90	78.4	Intron-flanking	95.9	0.9991
ACTB	Fw: ACAGAGCCTCGCCTTTGC Rev: CGCGGCGATATCATCATCCA	76	86.9	Intron-flanking	95.2	0.9998
GAPDH	Fw: GAGAAGGCTGGGGCTCATTT Rev: TGATGACCCTTTTGGCTCCC	46	79.4	Intron-spanning	97.8	0.9999

Regression coefficients were determined by performing least squares linear regression to the average Cq values of technical replicates. Base pairs, bp.

### RNA isolation and quality control

Cancer and normal cell lines were cultured and cells were collected from three biological replicates for RNA extraction. The purity and concentration of RNA samples were determined with NanoDrop, and the integrity and genomic DNA contamination were assessed by performing agarose gel electrophoresis (Supplementary Fig. S3). The characteristic rRNA bands 28S and 18S were visualized as two distinct bands without any evident degradation products or genomic DNA contamination. The lack of any considerable degradation together with the presence of the two characteristic ribosomal RNA bands confirm the good quality of all RNA preparations. In addition, we have also checked the UV–Vis spectrum of the preparations. The 260/280 ratios were in the range from 2.02 to 2.11 indicating absence of protein contamination (Supplementary Table S1).

### Optimization of the RT-qPCR conditions

**Figure 1:**
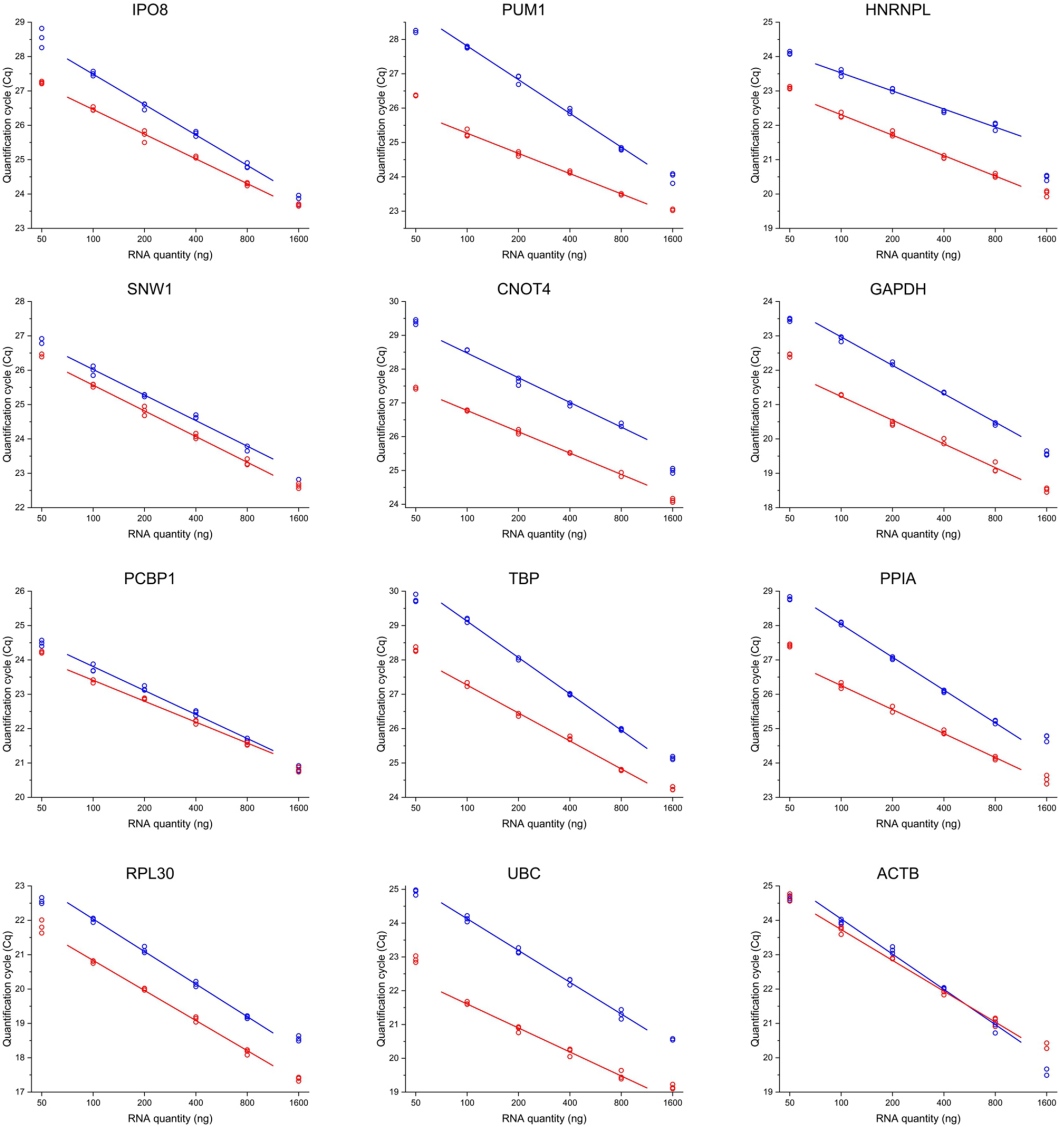
Optimization of the reverse transcription conditions. The graph shows Cq values from performing qPCR measurements of RNA dilution series comparing the Maxima First Strand cDNA Synthesis Kit for RT-qPCR (red lines) and the High-Capacity cDNA Reverse Transcription Kit (blue lines). Three technical replicates for both kits at each concentration point are marked as hollow circles. Least squares linear regression was performed to the average of the technical replicates in the range of RNA amount from 100 to 800 ng per reaction. Individual graphs were created with OriginPro 2018 (OriginLab Corp.) and the figure was assembled using CorelDRAW Graphics Suite 2020 (Corel Corporation).

The total RNA concentration, along with the priming strategy and the enzyme heavily affect the performance of the reverse transcription reaction^44–46^. Two commercially available kits—Maxima First Strand cDNA Synthesis Kit for RT-qPCR and High-Capacity cDNA Reverse Transcription Kit—were compared using RNA from the same series of 6 point fourfold dilutions. To compare the results obtained from different human cell lines, it is of utmost importance to work within the linear concentration range of the RT reaction. In case of both kits and all target genes, linearity was confirmed within the range of RNA amount between 100 and 800 ng per reaction (Fig. 1). We found, however, that the most and least concentrated point fell out of the linear range. Least squares linear regression was performed for the average of the technical replicates within the aforementioned range of 100–800 ng RNA. The slope of the line was found to be moderately steeper in case of the High-Capacity cDNA Reverse Transcription Kit, indicating better sensitivity, however the Cq values were considerably lower in most cases using the Maxima First Strand cDNA Synthesis Kit for RT-qPCR, which implies more efficient RT reaction (Fig. 1). As illustrated by the results, both kits are applicable for our study. For further experiments, the Maxima kit was selected and 200 ng total RNA was introduced to each reaction.

Determination of PCR efficiency is important for accurate quantification of target genes, moreover, it is paramount for selecting reference genes. High PCR efficiency is often correlated with robust and precise qPCR methods. Two approaches exist for PCR efficiency determination: serial dilutions with cDNA and serial dilutions with standard DNA templates. The advantage of the latter method is that a broader concentration range can be analyzed, however, the matrix effect of the cDNA sample is not taken into consideration. Based on these facts, we decided to determine the efficiency values from serial dilutions of PCR products (Supplementary Fig. S4), then the results were validated with measurements conducted with cDNA templates (Supplementary Table S2). The Cq values were plotted against the logarithm of template concentration, least squares linear regression was performed for the average of the technical replicates. Efficiency was determined from the slope of the regression lines. Efficiency values were higher than 95% for all genes except for IPO8 for which it was 92.5% (Table 3).

To assess the effect of various parameters of the quality of the initial RNA samples on the relative expression of the candidate reference genes, a statistical model was applied. A general linear model was built for each candidate reference gene as the dependent variable, using the cell line as a categorical predictor and the 260/280 and 260/230 ratios and the yield of the RNA preparations as continuous predictors. As an assumption of the model, the homogeneity of the variances was tested with Cochran’s C test. The result of the tests of significance along with the tests for the homogeneity of variances is available as Supplementary Dataset 3. The Cochran’s C test did not indicate inhomogeneity of variances. While the effect of the cell line studied was highly significant as characterized with p values of 0.000024 or less, the effect of neither the RNA quality parameters nor the yield of RNA proved to be significant on the relative expression of any candidate reference genes. The result of the analysis argues for RNA samples with appropriate purity and also demonstrate that the differences in the yield of RNA extraction were successfully eliminated by measuring equally 200 ng total RNA in each reverse transcription reaction.

### Reference gene expression stability

**Figure 2:**
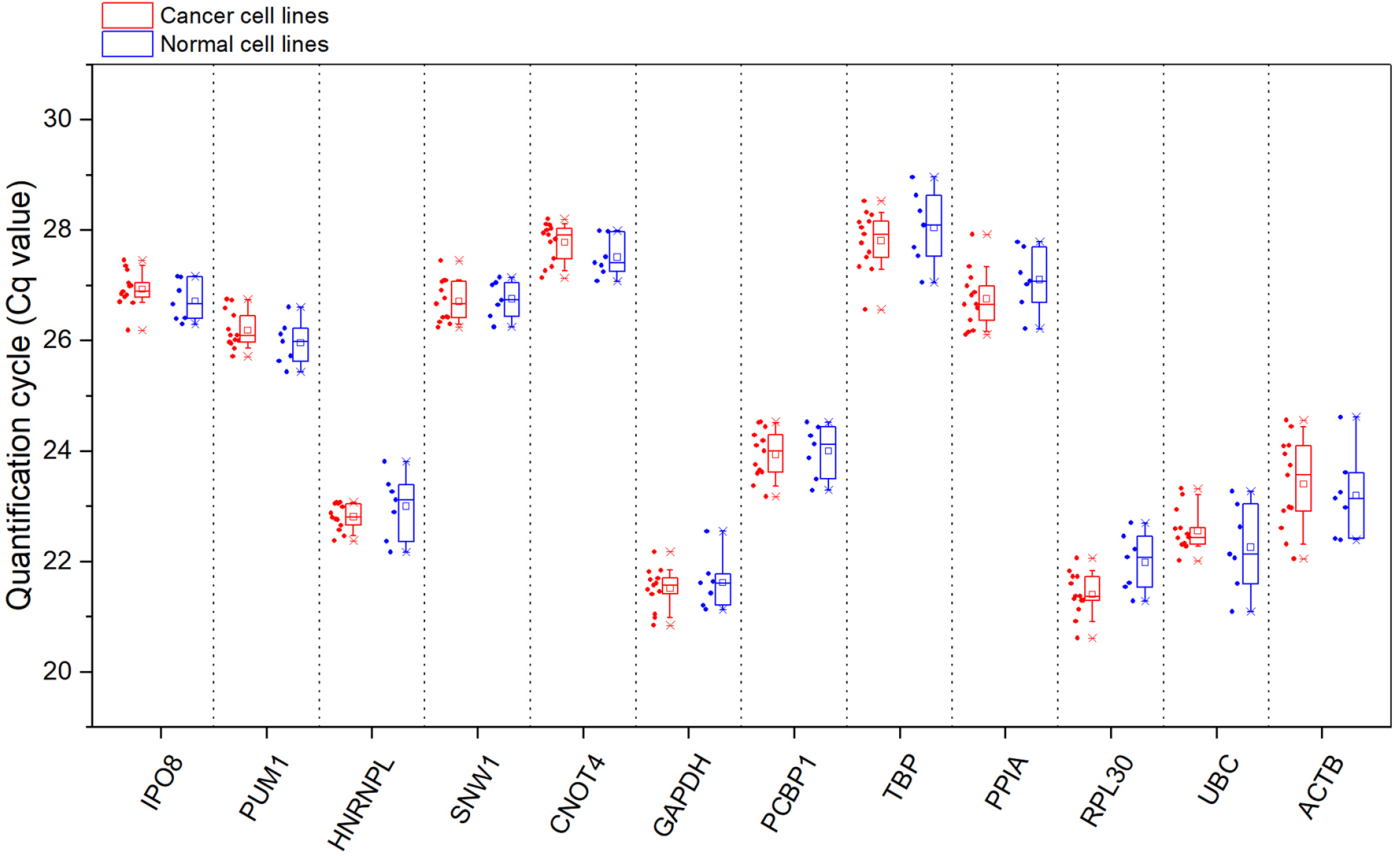
Cq values of twelve candidate reference genes in human cancer (red) and normal (blue) cell lines. Each dot represents the average Cq value of three biological replicates for each cell line. Candidate reference genes are arranged according to the comprehensive ranking in a decreasing order of expression stability. The boxes represent the data between the 25th and 75th percentile, while whiskers show the data range from the 10th to the 90th percentile. The minimum and maximum values are depicted as ‘x’. The average values are represented as squares and the median values are illustrated as lines. This graph was created with OriginPro 2018 (OriginLab Corp.).

The Cq values of the reference genes determined for both cancer and normal cell lines are shown in Fig. 2. HEK293, MRC-5, HUVEC/TERT2, HMEC, HFF-1, HUES 9, XCL-1 are included in our set of normal cell lines, while the group of cancer cell lines consists of HeLa, MCF-7, A-549, K-562, HL-60(TB), HT-29, MDA-MB-231, HCT 116, U-937, SH-SY5Y, U-251MG, MOLT-4 and RPMI-8226. All Cq values were distributed within 20 and 30 cycles. Cq values for ACTB and UBC exhibited the highest variation. In contrast, IPO8, PUM1 and—in case of cancer cell lines—HNRNPL showed the lowest variation. The Cq values for SNW1 and CNOT4—the novel candidate reference genes suggested by us—were also distributed within a relatively small range.

The stability of the reference genes was evaluated with GeNorm^24^, NormFinder^41^ and BestKeeper^42^ software and the Comparative ΔCt method^43^. The BestKeeper panel of RefFinder online tool was used instead of the BestKeeper Excel tool since the latter cannot handle more than 10 reference genes. However, RefFinder cannot take the PCR efficiency into consideration. These software products use different methods to determine the expression stability of the reference genes. It is common in these software products that a stability value is calculated for each gene and ranks are assigned based on the ascending order of these values. The comprehensive ranking of the expression stability of the reference genes is calculated as the geometric mean of the individual ranks given by the software. The ranks and characteristic stability parameters are summarized in Table 4. The analysis was conducted separately on all data, on the data from cancer cell lines and on the data from normal cell lines.

**Table 4:** Ranking of candidate reference genes.

Comprehensive rank	Gene symbol	GeNorm		Comparative ΔCt		NormFinder		BestKeeper		Geometric mean
		Rank	M value	Rank	Mean StdDev	Rank	Stability value	Rank	Stability value	
**All cell lines**										
1	IPO8	1	0.381	3	0.555	1	0.205	3	0.336	1.73
2	PUM1	2	0.384	4	0.568	4	0.222	1	0.326	2.38
3	HNRNPL	3	0.386	1	0.538	3	0.215	6	0.353	2.71
4	SNW1	4	0.407	2	0.545	2	0.206	4	0.344	2.83
5	CNOT4	5	0.428	5	0.597	5	0.266	5	0.345	5.00
6	GAPDH	10	0.547	7	0.652	**6**	**0.268**	2	0.328	5.38
7	PCBP1	6	0.45	6	0.612	7	0.273	7	0.394	6.48
8	TBP	7	0.477	9	0.678	**9**	**0.342**	11	0.483	8.89
9	PPIA	8	0.504	8	0.657	8	0.308	10	0.471	8.46
10	RPL30	9	0.523	10	0.701	11	0.366	8	0.406	9.43
11	UBC	11	0.587	11	0.777	10	0.362	9	0.457	10.22
12	ACTB	12	0.636	12	0.911	12	0.503	12	0.679	12.00
**Cancer cell lines**										
1	HNRNPL	1	0.333	1	0.475	1	0.160	1	0.275	1.00
2	IPO8	2	0.346	3	0.535	2	0.212	5	0.322	2.78
3	PUM1	3	0.363	4	0.539	4	0.215	4	0.32	3.72
4	SNW1	5	0.416	2	0.533	3	0.213	8	0.368	3.94
5	CNOT4	4	0.393	5	0.564	6	0.257	6	0.332	5.18
6	GAPDH	10	0.523	7	0.616	**5**	**0.245**	3	0.31	5.69
7	RPL30	7	0.467	9	0.627	11	0.321	2	0.308	6.10
8	UBC	6	0.437	6	0.599	7	0.260	7	0.355	6.48
9	PCBP1	9	0.503	8	0.623	8	0.299	9	0.394	8.49
10	TBP	8	0.487	10	0.640	**10**	**0.312**	10	0.447	9.46
11	PPIA	11	0.539	11	0.644	9	0.308	11	0.458	10.46
12	ACTB	12	0.595	12	0.904	12	0.510	12	0.716	12.00
**Normal cell lines**										
1	IPO8	1	0.223	1	0.495	1	0.149	3	0.318	1.32
2	SNW1	2	0.236	3	0.515	2	0.187	1	0.29	1.86
3	PUM1	3	0.26	2	0.508	**5**	**0.218**	4	0.323	3.31
4	CNOT4	9	0.433	5	0.552	**3**	**0.203**	2	0.301	4.05
5	PCBP1	6	0.368	4	0.544	4	0.214	6	0.386	4.90
6	PPIA	5	0.349	7	0.570	6	0.282	7	0.422	6.19
7	HNRNPL	4	0.327	6	0.568	7	0.286	9	0.455	6.24
8	GAPDH	10	0.481	9	0.678	8	0.306	5	0.368	7.75
9	RPL30	7	0.387	8	0.632	9	0.318	8	0.437	7.97
10	TBP	8	0.407	10	0.685	10	0.374	10	0.533	9.46
11	ACTB	11	0.554	11	0.884	11	0.464	11	0.553	11.00
12	UBC	12	0.622	12	0.967	12	0.554	12	0.617	12.00

The comprehensive rank is generated based on the geometric mean of ranks. The best combinations of reference genes as suggested by NormFinder are shown in bold.

The comprehensive ranking based on the analysis conducted on the expression data of all cell lines identified IPO8 as the most stable reference gene, followed by PUM1, HNRNPL, SNW1 and CNOT4 in order. Moreover, these five genes were also among the best 5 genes as suggested by GeNorm, NormFinder, BestKeeper and the Comparative ΔCt individually—except for HNRNPL, which was ranked 6th by BestKeeper. For our set of cancer cell lines, HNRNPL was found to be the most stable reference gene as determined by all evaluation methods consistently. HNRNPL was followed by IPO8, PUM1, SNW and CNOT4 in a decreasing order of expression stability according to the comprehensive ranking. IPO8, PUM1 and SNW1 were included in the top 5 ranking genes suggested by all evaluation methods—with the only exception of SNW1 in case of BestKeeper, which was given a rank of 8. CNOT4 gained ranks between 4 and 6 by all four methods. In case of our set of normal cell lines, IPO8, SNW1, PUM1 and CNOT4 were identified as the most stable reference genes according to the comprehensive ranking, in order of decreasing expression stability. Similarly to the analysis on the expression data for cancer cell lines and all cell lines, these four genes were included in the best 5 genes suggested by all methods individually—except that CNOT4 was 9th in the ranking given by GeNorm.

NormFinder also identifies the best combination of two genes, which are shown in bold in Table 4. Theses combinations of genes, however, are not comprised of the top ranking genes. In addition, GeNorm also determines the optimal number of reference genes to be used in a study investigating gene expression (Supplementary Fig. S5 for all cell lines, Supplementary Fig. S6 for cancer cell lines and Supplementary Fig. S7 for normal cell lines). This analysis is based on the pairwise variation V between two sets of genes that contain increasing number of genes. As suggested by the original article describing GeNorm^24^, inclusion of no more genes is necessary when the V value is below 0.15. Our analysis indicate that using two genes as reference is sufficient for gene expression analysis both for cancer cell lines and normal cell lines and also for all cell lines investigated.

### Evaluation of the stability of the reference genes upon serum starvation

Serum starvation constitute one of the most frequently performed cell culture condition that can easily be standardized, since it actually means that a limited set of components is present in the medium^47^. Therefore, we have chosen serum starvation as a specific cell culture condition for our study because our major aim was to identify novel reference genes under well-standardized and widely used conditions, also including many different cell lines. For other conditions (for instance drug treatments, addition of specific factors, mutagenic conditions, etc.), a large variation can be expected, since the quality of the medium, the serum and the characteristics and concentration of the additional components vary considerably in different laboratories. Therefore, these distinct conditions are not within the context of our present study.

**Figure 3:**
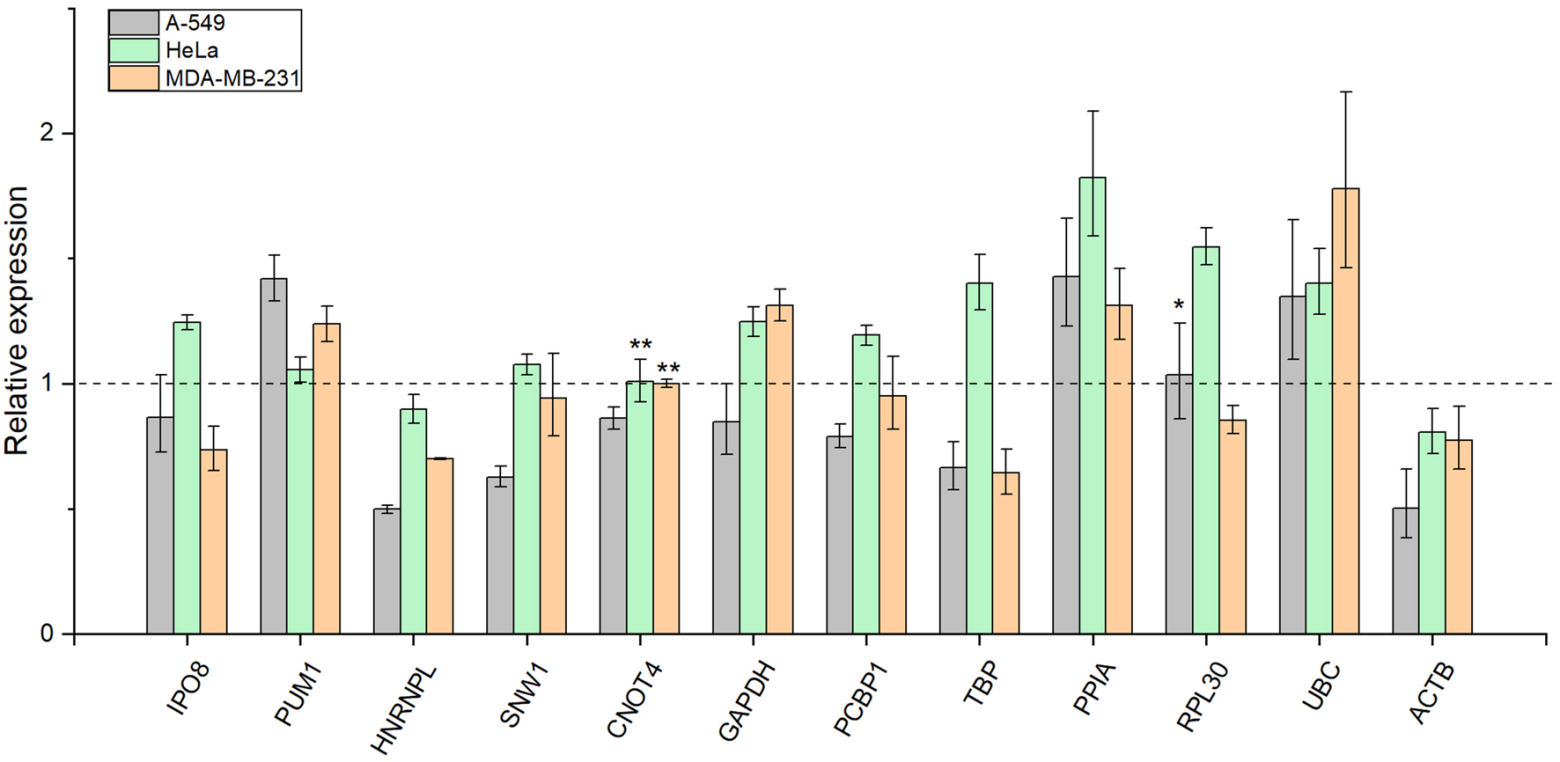
Relative expression of the candidate reference genes upon serum starvation. Relative expression values of serum starved cell lines can be compared to the average relative expression of the non-treated biological replicates selected as control and set to 1, which is represented by a dotted line. Error bars show standard deviation of three biological replicates (n = 3) for each cell line. The number of asterisks indicate increasing possibility that gene expression remains constant upon serum starvation. *p > 0.8, **p > 0.95 as calculated by the CFX Maestro software. This graph was created with OriginPro 2018 (OriginLab Corp.).

Three human cancer cell lines (A-549, MDA-MB-231 and HeLa) were submitted to serum starvation as these cell lines survive but cease to proliferate under serum-free condition according to the literature^48^. The relative expression of the candidate reference genes in three serum-starved biological replicates were compared with three biological replicates under normal conditions. Figure 3 shows the relative expression data for the reference genes. The average relative expression values for each cell line under normal conditions were set to 1. We sought to identify genes with invariable expression upon such conditions, thus genes with relative expression values close to 1 are ideal to compare gene expression in cell cultures undergoing serum starvation. The relative expression values with the range of error and associated p values are summarized in Supplementary Table S3. According to this analysis, we identify CNOT4 as the most stable reference gene under these experimental conditions. The expression of RPL30 was also found to be stable in A-549 cell line upon starvation. The expression of HNRNPL and ACTB decreases upon serum starvation in all three cell lines investigated. In contrast, the expression of PPIA and UBC increases in all three cell lines. The expression of other genes included in our candidate reference gene set is altered in different directions depending on the cell line studied. For example, the expression of PPIA increases in A-549 and MDA-MB-231 cells, however, decreases in Hela cells upon serum starvation. The magnitude of change also varies considerably depending on the cell line investigated.

## Discussion

**Figure 4:**
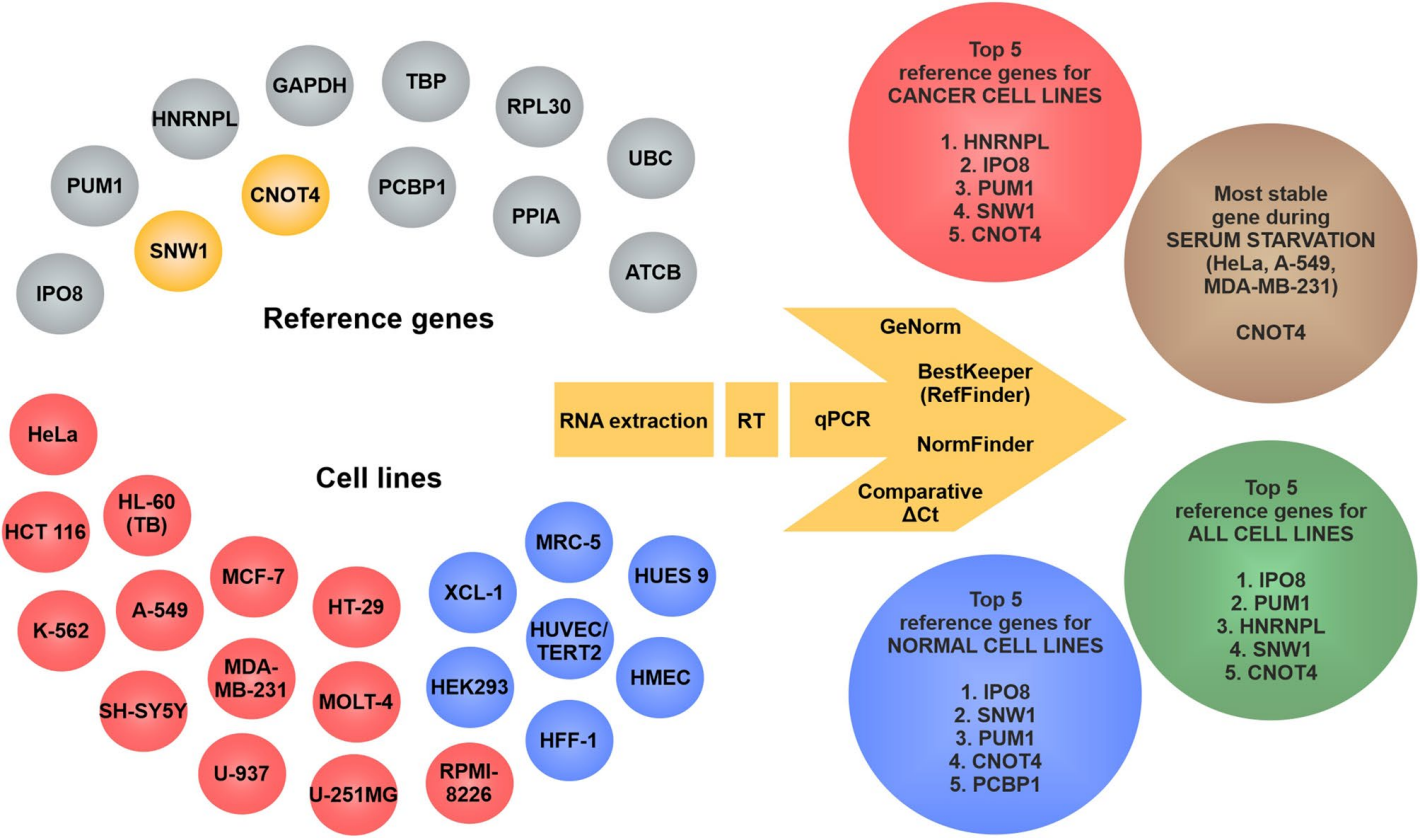
Schematic figure of the overall experimental design and the results of this study. In the upper left corner the investigated candidate reference genes are shown in grey except for the novel reference genes SNW1 and CNOT4, which are shown in yellow. The investigated cell lines are depicted in the lower left corner. Cancer cell lines are colored red, while normal cell lines are colored blue. On the right side the top-ranking reference genes are illustrated for cancer cell lines in red circle, for normal cell lines in blue circle and for all cell lines in green circle. The most stable gene upon serum starvation is shown in brown circle. The methods used for performing the experiments and the software used for the evaluation of the results are depicted as a yellow arrow. This graph was created with CorelDRAW Graphics Suite 2020 (Corel Corporation).

We investigated twelve candidate reference genes with RT-qPCR in human cancer and normal cell lines extensively used in scientific research. Figure 4 shows the overall experimental design and summarizes the results of the present study. We included two novel genes—SNW1 and CNOT4—in this study based on an evaluation we performed on the RNA HPA cell line gene data available in The Human Protein Atlas^39,40^. The data suggested low variability in the expression of these genes among cell lines included in the database, however, SNW1 and CNOT4 have never been suggested as reference genes in the literature. Additionally, we decided to investigate HNRNPL and PCBP1 as suggested by Jo et al.^34^, furthermore, other widely used genes (IPO8, PUM1, GAPDH, TBP, PPIA, RPL30, UBC, ACTB) were also included in our study. To perform a comprehensive study we sought to encompass commonly used human cancer and normal cell lines. Moreover, a commonly used technique, serum starvation was also applied to investigate the expression of the candidate reference genes under such experimental condition.

The results were evaluated using the available software (GeNorm, NormFinder, BestKeeper as a panel of RefFinder and the Comparative ΔCt method) and a comprehensive ranking was calculated to arrange the candidate reference genes in an increasing order of the stability of their expression regarding all cell lines, and specifically cancer and normal cell lines. Each method has its advantages and limitations. The GeNorm and the Comparative ΔCt methods are susceptible to favor coregulated genes, since the ranks given by these methods are based on the correlation of gene expression between samples. BestKeeper also employs correlation analysis, nonetheless, ranks are assigned mostly based on the standard deviation of the Cq values, thus BestKeeper analysis is essentially devoid of such error. NormFinder, in contrast, utilizes a statistical model estimating not only intra- but also inter-group variation taking into account sample groups. As the methods use a different approach to determine the ranking, the results generated by the software cannot be compared directly on the basis of the stability values, thus the final ranking was calculated as the geometric mean of the individual ranks given by the software products.

IPO8, PUM1 and HNRNPL were among the three most stable genes for our set of cancer cell lines and also for all cell lines investigated. IPO8 and PUM1 ranked 1st and 3rd for our set of normal cell lines. Novel candidate reference genes SNW1 and CNOT4 were identified as the 4th and 5st most stable genes for all cell lines and also for cancer cell lines, while ranked 2st and 4th in case of normal cell lines, respectively. Importantly, we also find that many commonly used reference genes perform poorly and show large variations among the different cell lines (ACTB, UBC, RPL30, PPIA, TBP).

The expression of the majority of our candidate reference genes change upon serum starvation. The direction and magnitude of the change depends on the cell line under investigation. Based on our results, we propose CNOT4 as a stable reference gene for expression studies using cancer cell lines HeLa and MDA-MB-231 undergoing serum starvation. The use of CNOT4 as reference is also possible for A-549 cells as the change in relative expression is as small as 14%. SNW1 may also be used as an appropriate reference gene for HeLa and MDA-MB-231 upon serum starvation, since the change in relative expression is less than 10%.

In conclusion, we propose the use of IPO8, PUM1, HNRNPL, SNW1 and CNOT4 as reference genes in studies comparing gene expression between different cancer and/or normal cell lines. In addition, we propose CNOT4 and SNW1 as stable reference genes for investigating gene expression in cell lines undergoing serum starvation. It should be noted, however, that our results apply to our set of cell lines and extending these findings to other cell lines not investigated in this study should always be accomplished by careful experimental validation.

## Methods

### Selection of cell lines

With the intention to select popular cancer cell lines, literature available in the PubMed database was searched for the members of NCI-60 with additional cell lines, JFCR39, KuDOS 95 and LL-100 cell line panels. In addition, Hela, HEK293 and SH-SY5Y were also included. The search included all synonyms for the cell lines available in the Expasy Cellosaurus database. The search was performed only in the title and abstract of the articles. The cell lines were arranged in decreasing order of the number of search results. The names of the cell lines, accession numbers (RRID) and the related diseases according to Cellosaurus, the number of articles found, the search strings and the cell line panels in which the cell lines are included are presented as Supplementary Dataset 1. In case of a few cell lines with short names, the search was not conclusive as the search results contained mostly irrelevant articles (BE [Human colon carcinoma], KB, MDA-N, PA-1, St-4, SEM, VAL, Ri-1, HC-1, HH [Human lymphoma], DEL, KG-1, CMK, SET-2), thus the result were omitted from the table.

### Cell culture

Cell lines A-549, HL-60(TB), HCT 116, HT-29, K-562, MCF-7, MDA-MB-231, MOLT-4, MRC-5, RPMI-8226 were obtained from the National Cancer Institute’s Developmental Therapeutics Program (National Institutes of Health). HeLa (CCL-2), HEK293 (CRL-1573), U-937 (CRL-1593.2), SH-SY5Y (CRL-2266) and the human foreskin fibroblast cell line HFF-1 (SCRC-1041) were purchased from ATCC. MRC-5 and HUVEC/TERT2 were a generous gift from József Tóvári. The XCL-1, induced pluripotent stem cell line reprogrammed from CD34+ cord blood cells by episomal vectors, were purchased from XCellScience (Novato, CAXIP-001-1V). HMEC cells immortalized with TERT were obtained from the Francis Crick Institute Cell Services Department. The HUES9 human pluripotent stem cell line was kindly provided by Douglas Melton (HHMI). A-549, HCT 116, HEK293, HeLa, HL-60(TB), HT-29, K-562, MCF-7, MDA-MB-231, MOLT-4, RPMI-8226, SH-SY5Y, U-251MG and U-937 cells were cultured in Roswell Park Memorial Institute (RPMI) 1640 medium (Gibco 72400-021) supplemented with 10% heat-inactivated fetal bovine serum (FBS) (Gibco 10500064) and 1% Penicillin Streptomycin (Gibco 15140-122). MRC-5 cells was cultured in Dulbecco’s Modified Eagle Medium (DMEM) (Gibco 11995-065) supplemented with 20% FBS and 1% Penicillin Streptomycin. HUVEC/TERT2 cells was cultured in EBM-2 Endothelial Cell Growth Basal Medium-2 (Lonza 00190860) supplemented with components from the EGM-2 Endothelial SingleQuots Kit (Lonza CC-4176). HMEC cells were cultured in MEGM Mammary Epithelial Cell Growth Medium BulletKit (Lonza CC-3150). XCL-1 and HUES 9 cells were maintained on Matrigel (Corning) coated six well plates in mTeSR medium (Stemcell Technologies). HFF-1 cells were maintained on gelatin (Sigma) coated plates in DMEM-glutamax medium completed with 10% FBS (Thermo Fisher Scientific). All cell lines were cultivated at 37 °C in a humidified incubator with 5% CO2 atmosphere. All cell cultures were free of mycoplasma as determined by PCR. Adhesion cell lines were passaged when the culture reached 40–50% confluency to avoid contact inhibition. Suspension cell lines were passaged every 2–3 days. For RNA extraction cells were collected after 2 days of passage.

### RNA extraction, determination of concentration, purity and integrity

Suspension cells and trypsinized adhesive cells were centrifuged at 250*g* for 5 min in Eppendorf MiniSpin centrifuge, washed twice with Phosphate buffered saline (Sigma), and resuspended in RLT Plus buffer (Qiagen RNeasy Plus Mini Kit) with 1% beta-Mercaptoethanol (Merck) and lysed with sterile glass beads by vortexing for one minute. Samples were kept at − 20 °C until further processing. RNA was isolated using Qiagen RNeasy Plus Mini Kit according to the manufacturer’s recommendations. DNA was digested on-column with RNase-Free DNase Set (Qiagen) according to the manufacturer’s recommendations. RNA was eluted with 50 µl nuclease-free water (Ambion). The concentration and purity of the samples were determined with NanoDrop ND-2000. The integrity of RNA and genomic DNA contamination were assessed by performing agarose gel electrophoresis with 1% agarose (Sigma A9539) and TBE running buffer using equally 600 ng RNA. GeneRuler 1 kb Plus DNA Ladder (Thermo Scientific SM1331) was loaded as marker and gel loading dye (New England Biolabs B7024S) was used. Gel Doc XR + Imager (Bio-Rad) was used for imaging. RNA samples were kept at − 80 °C.

### Selection of candidate reference genes and primer design

The RNA HPA cell line gene data was downloaded from The Human Protein Atlas and imported to Microsoft Office Professional Plus Excel 2013. It contains transcripts per million ("TPM"), protein-coding transcripts per million ("pTPM") and normalized expression ("NX") data for 69 cell lines based on The Human Protein Atlas version 20.1 and Ensembl version 92.38^39,40^. The data was filtered based on the number of cell lines with available data and only those with at least 30 cell lines were included in our analysis. The mean and standard deviation of the normalized expression and the coefficient of variation (CV) were calculated for each gene. The genes were ranked according to the CV values in an ascending order. The calculated data is available as Supplementary Dataset 2. Literature available in the PubMed database was searched for commonly used reference genes, for the reference genes suggested by Jo et al.^34^ and for the top three genes based on the ranking with the keywords “reference gene” or “reference genes” and “human” and the symbol of the gene. The search was performed only in the title and abstract of the articles. Only those genes appearing in at least 10 articles and the genes selected in this study are included in Table 2.

Twelve reference genes were selected for our study. The sequences of all existing transcript variants for the targets in the NCBI Gene database were downloaded from NCBI Reference Sequences (RefSeq) database. Primers were designed for amplification of the common sequence of all variants. Regions with single nucleotide polymorphism, deletions or insertions with at least 1% prevalence according to the 1000 Genomes MAF project were excluded as indicated by the NCBI's Variation Viewer. The NCBI primer designing tool was used to design primer pairs. Preferentially primers located on an exon-exon junction (intron-spanning primer design) or on two consecutive exons separated by an intron (intron-flanking primer design) were selected whenever possible. PCR product length was limited to 120 base pairs (bp). The melting temperatures of the primers were set to be in the range of 60–63 °C. Specificity was investigated with BLAST with the following parameters: at least 5 total mismatches to unintended targets, including at least 3 mismatches within the last 5 bps at the 3' end. Targets with more than 6 mismatches were ignored for the specificity check.

For each target three or four primer pairs were designed and ordered from Sigma–Aldrich with desalting purification. All primer pairs were tested experimentally with temperature gradient qPCR. Those primer pairs were selected for which the PCR product was specific as indicated by melting curve analysis and agarose gel electrophoresis, and the annealing temperature for all targets were identical, and the Cq values were the lowest as compared to the other primer pairs for the given target.

### RT-qPCR

For reverse transcription the Maxima First Strand cDNA Synthesis Kit for RT-qPCR (Thermo Scientific K1642) was used with 200 ng RNA introduced to the reaction, unless otherwise noted. The RT reaction was performed in Applied Biosystems GeneAmp PCR system 2700. cDNA samples were kept at − 20 °C until further processing. The qPCR reaction was performed in 10 µl final volume using MyTaq HS Mix (Bioline BIO-25046), Evagreen dye (Biotium 31000), nuclease-free water, cDNA template, and appropriate primers. Primers were obtained from Sigma with desalting purification in a dry format and dissolved in nuclease-free water according to the recommendation to make 100 µM solutions. The concentration of the primer solutions were checked by NanoDrop to adjust the final concentration in the PCR reaction to 500 nM. In each qPCR reaction 0.31 µl cDNA sample was used. For every sample and every target gene, three technical replicates were used. Two technical replicates of no template control (NTC) reaction were measured on each plate. No reverse transcription control (NRT) were measured randomly for 25% of the samples. NRT samples were prepared from the RNA samples without the RT enzyme and reaction mix. The difference between the Cq values of the NRT/NTC and the samples were higher than 10 in most cases, and higher than 5 in all cases.

Clear Hard-Shell 96-Well PCR Plates (Bio-Rad) and Microseal ‘B’ PCR Plate Sealing Film (Bio-Rad) were used. Thermal cycling and detection was performed in CFX96 real-time PCR detection system (Bio-Rad). Thermal cycling conditions were set as follows: 95 °C for 5 min followed by 50 cycles of 95 °C for 30 s and 63 °C for 30 s. After amplification, melting curve analysis was performed from 60 to 95 °C with an increment of 0.5 °C every 5 s, unless otherwise noted.

### Assessment of the RT reaction

Two reverse transcription kits were compared: Maxima First Strand cDNA Synthesis Kit for RT-qPCR (Thermo Scientific K1642) and High-Capacity cDNA Reverse Transcription Kit (Applied Biosystems 4368814). The kits were used according to the manufacturer’s recommendations and for the Maxima First Strand cDNA Synthesis Kit for RT-qPCR, the RT reaction was performed at 65 °C for 30 min. A series of 6 point twofold dilutions was prepared and introduced to the RT reaction with the starting concentration of 1600 ng/µl.

### Determination of PCR efficiency

cDNA derived from three biological replicates of HCT 116 cell line was mixed and amplified in qPCR reactions for each target gene. For each PCR product agarose gel electrophoresis with 2% agarose and TAE buffer was performed and purified from the gel with NucleoSpin Gel and PCR Clean-up (Macherey–Nagel 740609). The concentration of the purified PCR products was measured with NanoDrop and a series of 7 point tenfold dilution was prepared and introduced to the qPCR reaction in the range of concentration from 100 to 0.0001 fg/µl. Three technical replicates were applied for each target gene and every concentration point. The Cq values were plotted against the logarithm with base 10 of the concentration and the slope of the curves and regression coefficients were determined and the PCR efficiency values were calculated with the formula E (%) = [10^(1/−slope)−1^] × 100%. The PCR efficiency values obtained from the measurements with PCR products were used for further calculations.

PCR efficiency determination was also performed using cDNA dilutions for 5 target genes (IPO8, PUM1, SNW1, GAPDH, PPIA) to validate the results obtained from the measurements with PCR products. cDNA derived from three biological replicates of HCT 116 cell line was mixed and a 6 point fourfold dilution series was prepared and three technical replicates were applied for each concentration point and target gene. The most concentrated PCR reaction contained 0.31 µl cDNA. The evaluation of the results was performed the same way as shown for the PCR products.

### Assessment of the specificity of PCR

The specificity of the PCR reaction was assessed with agarose gel electrophoresis and melting curve analysis. For each candidate reference gene PCR products using HCT 116 samples were analyzed with agarose gel electrophoresis with 2% agarose and TAE running buffer. For each gene 2–5 µl of PCR products were mixed with loading dye and loaded on the gel. GeneRuler 1 kb Plus DNA Ladder and GeneRuler 100 bp Plus DNA Ladder (Thermo Scientific SM0321) were used as markers. Gel Doc XR + Imager was used for imaging. Melting curve analysis was routinely performed after amplification. Technical replicates of qPCR reactions were excluded in case of aspecific product formation according to the melting curve analysis.

### Determination of reference gene expression stability and data analysis

For data collection the CFX Maestro 2.0 (Bio-Rad) software was used (URL: https://www.bio-rad.com/en-us/product/cfx-maestro-software-for-cfx-real-time-pcr-instruments). The threshold value was set to 500 relative fluorescence unit (RFU) for every plate measured. Raw Cq values and relative expression values calculated by the CFX Maestro software were exported to Excel. For gene expression stability analysis, four methods were used: GeNorm, NormFinder software, the BestKeeper panel of the RefFinder web tool and the Comparative ΔCt method. GeNorm calculates the average pairwise variation of the expression of each target with all other candidate reference genes and the M value as the expression stability measure is generated for each target^24^. The most stable gene is characterized with the lowest M value. The software eliminates the gene with the highest M value in a stepwise fashion—that is, the target with the lowest expression stability—and recalculates the M values for the remaining genes. The software also estimates the optimal number of reference genes for normalization of gene expression using the pairwise variation V between two sets of genes that contain increasing number of genes. As a threshold value, 0.15 is recommended below which, the inclusion of more reference genes is not necessary. For the analysis with the GeNorm software, raw Cq values for the biological replicates were imported into the qBase + (Biogazelle) software and PCR efficiency values were considered. NormFinder employs a statistical model for the determination of inter- and intragroup variation between sample subgroups^41^. The expression stability value is calculated for each candidate reference gene based on the overall variation of gene expression. The lowest stability value indicates the reference gene with the most stable expression. The analysis with NormFinder was performed in Excel using the relative expression values calculated by the CFX Maestro software, in which the PCR efficiency values are considered. BestKeeper uses two approaches for the estimation of gene expression stability^42^. On one hand, descriptive statistics including standard deviation are calculated for each gene. On the other hand, the BestKeeper index is calculated as the geometric mean of the Cq values using the most stable genes, and subsequently the correlation between the index and each candidate reference gene is computed. Low standard deviation, as well as high correlation with the BestKeeper index implies stable genes expression characterized with a low stability value. For the analysis with BestKeeper the RefFinder web tool was used, as the maximum number of reference genes analyzed with BestKeeper Excel tool is limited to 10. The PCR efficiency values, however, cannot be considered with this tool. In the BestKeeper panel of the RefFinder tool, the ranking is mostly based on the standard deviation analysis^49^. For the Comparative ΔCt method, differences between the Cq values of every combination of two reference genes were calculated for every biological replicates^43^. The standard deviation of the Cq differences for every combination of two genes was calculated and the standard deviation values belonging to each reference genes were averaged. The M value from GeNorm, the stability value from NormFinder, the stability value from BestKeeper and the average standard deviation from the Comparative ΔCt method were used individually to order the reference genes based on a rank. Indicating the expression stability of the candidate reference genes, the final comprehensive rank was calculated as the geometric mean of the individual ranks.

Gel images were captured with Image Lab 4.1 software (Bio-Rad) (URL: https://www.bio-rad.com/en-hu/product/image-lab-software). Graphs were created with OriginPro 2018 (OriginLab Corp.) (URL: https://www.originlab.com/2018). CorelDRAW Graphics Suite 2020 (Corel Corporation) was used for creating figures from individual graphs (URL: https://www.coreldraw.com/en/product/coreldraw/).

For the serum starvation experiment, the p values were calculated by CFX Maestro software.

General linear models for testing significant effects of the parameters of RNA quality on the relative expression data were calculated with STATISTICA 10 (StatSoft Inc.) (URL: https://www.statistica.com/en/software/tibco-data-science-/-tibco-statistica).

## Supplementary Information

Supplementary Information 1.

Supplementary Information 2.

Supplementary Information 3.

Supplementary Information 4.

## Data Availability

The RNA HPA cell line gene dataset analyzed during the current study is available in The Human Protein Atlas, v20.proteinatlas.org/download/rna_celline.tsv.zip. Raw data is available upon request.
