# Supplementary material for: Identification of new reference genes with stable expression patterns for gene expression studies using human cancer and normal cell lines
Source: Sci Rep. 2021 Sep 30;11:19459. doi: 10.1038/s41598-021-98869-x (PMC8484624; doi:10.1038/s41598-021-98869-x)
Supplement: Supplementary file 4 — Supplementary Information 4. [file 41598_2021_98869_MOESM4_ESM.pdf]

Supplementary Information

A rational approach to select relevant reference genes for gene expression studies: Identification of new genes with stable expression patterns

Gergely Attila Rácz[1,2*], Nikolett Nagy[2,3], József Tóvári[4], Ágota Apáti[2], Beáta G. Vértessy[1,2*]

[1]Department of Applied Biotechnology and Food Sciences, Budapest University of Technology and Economics, Budapest, Hungary
[2]Institute of Enzymology, Research Center for Natural Sciences, Eötvös Loránd Research Network, Budapest, Hungary.
[3]Faculty of Science, Institute of Biology, Department of Biochemistry, Eötvös Loránd University, Budapest, Hungary
[4]Department of Experimental Pharmacology, National Institute of Oncology, Budapest, Hungary
*Corresponding authors

Correspondence and requests for materials should be addressed to Beáta G. Vértessy (email: vertessy@mail.bme.hu). Correspondence may also be addressed to Gergely Attila Rácz (racz.gergely@ttk.hu).

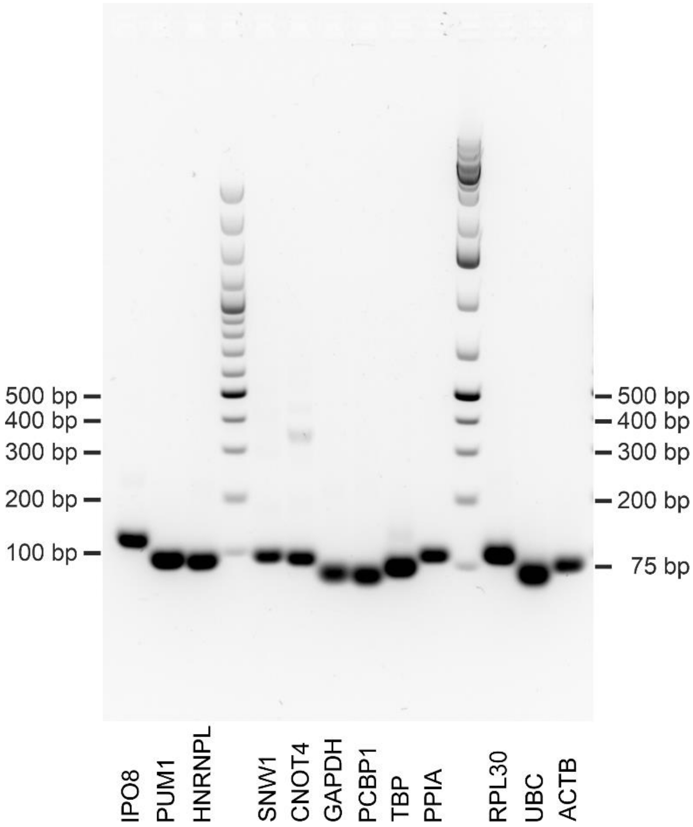

Supplementary Figure S1. PCR product quality control with agarose gel electrophoresis of PCR products. On the left side, GeneRuler 100 bp Plus DNA ladder, while on the right side GeneRuler 1 kb Plus DNA ladder was used as molecular-weight size marker. This image was captured with Image Lab 4.1 software (Bio-Rad) (refer to Methods for URLs).

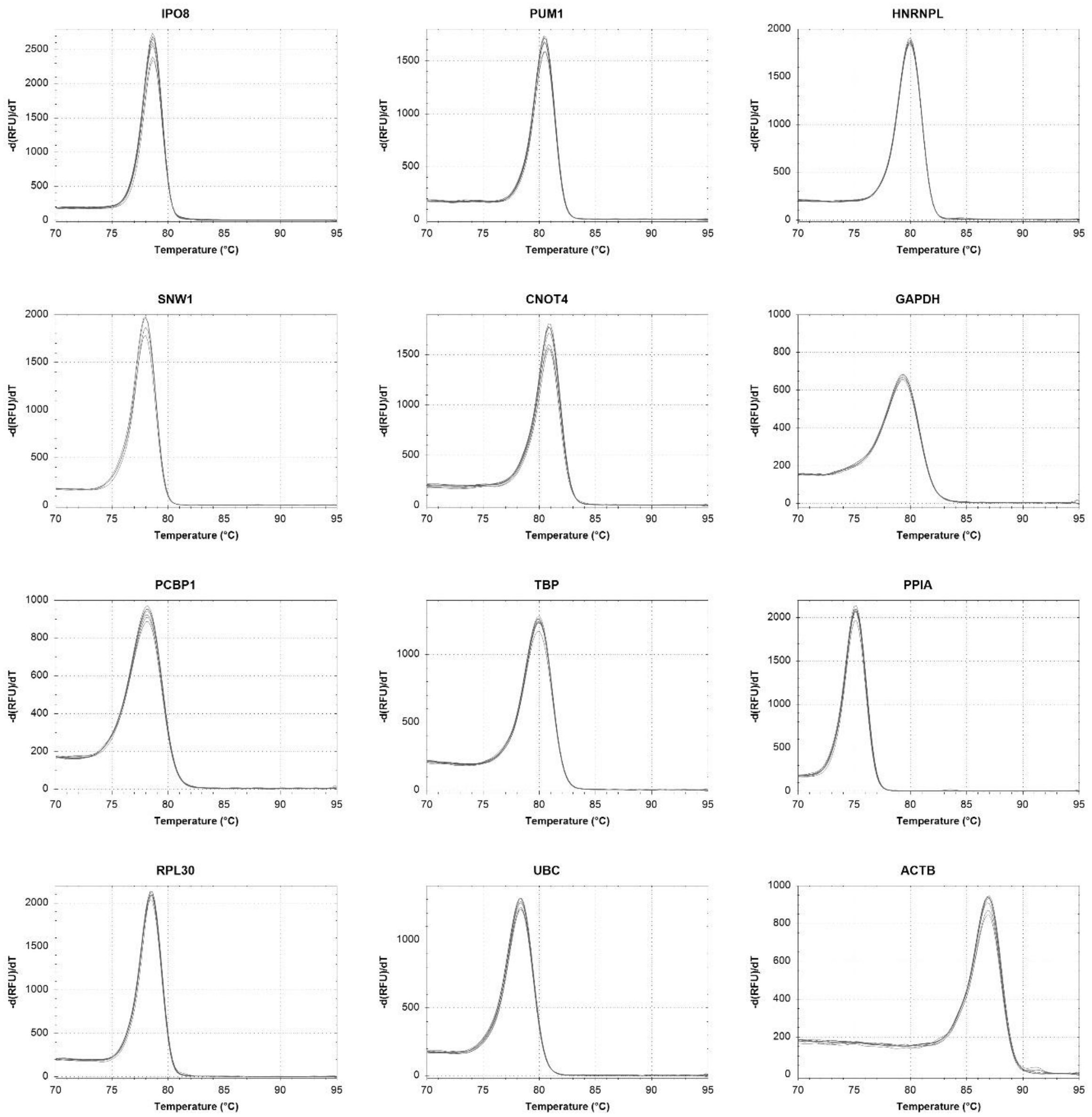

Supplementary Figure S2. Melting curve analysis of PCR products from 70 °C to 95 °C with an increment of 0.2 °C every 5 seconds. Individual graphs were created with CFX Maestro 2.0 software (Bio-Rad) and the figure was assembled using CorelDRAW Graphics Suite 2020 (Corel Corporation) (refer to Methods for URLs).

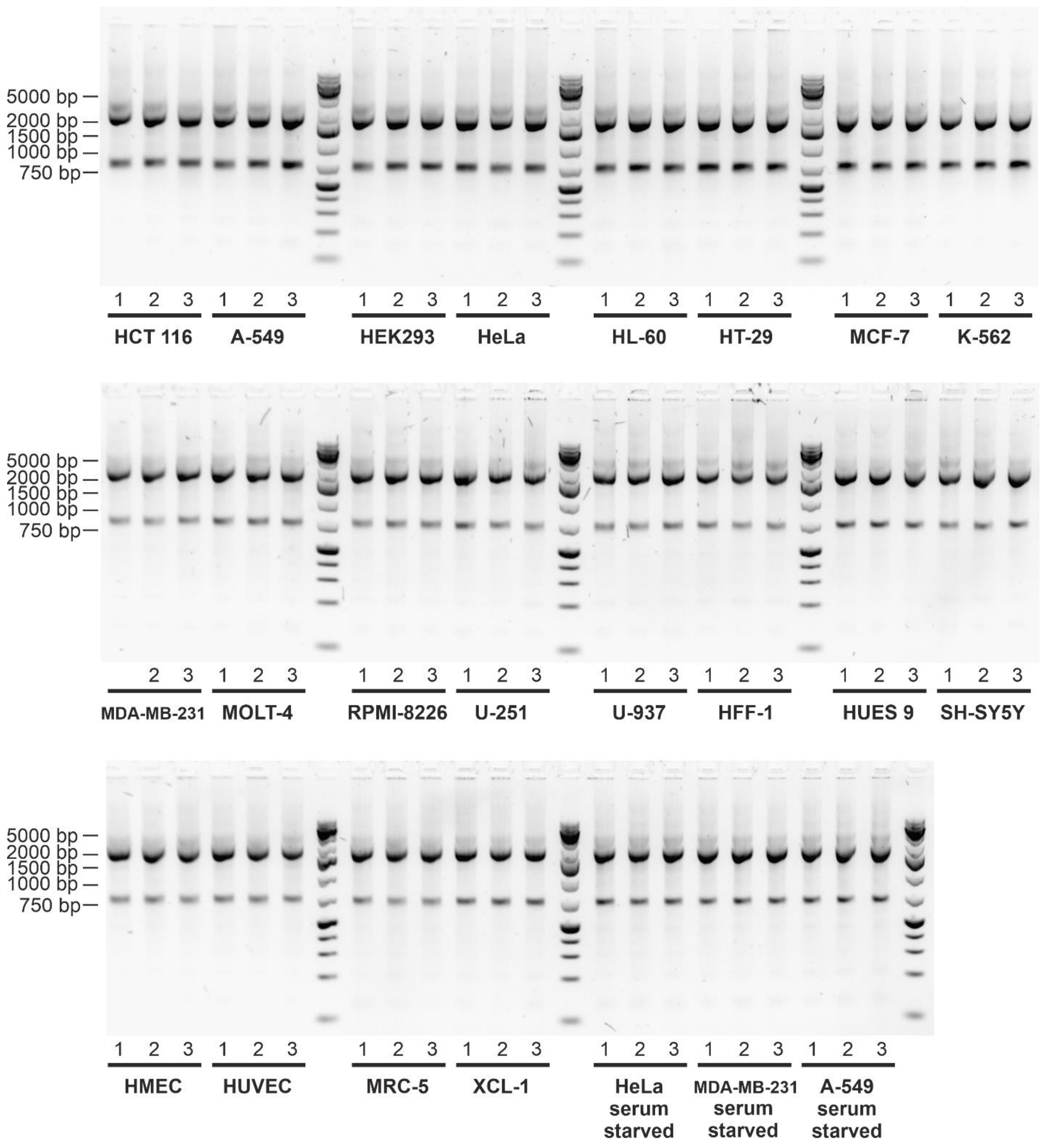

Supplementary Figure S3. RNA integrity assessment by performing agarose gel electrophoresis of 600 ng of each RNA sample. GeneRuler 1 kb Plus DNA ladder was used as molecular-weight size marker. This image was captured with Image Lab 4.1 software (Bio-Rad) (refer to Methods for URLs).

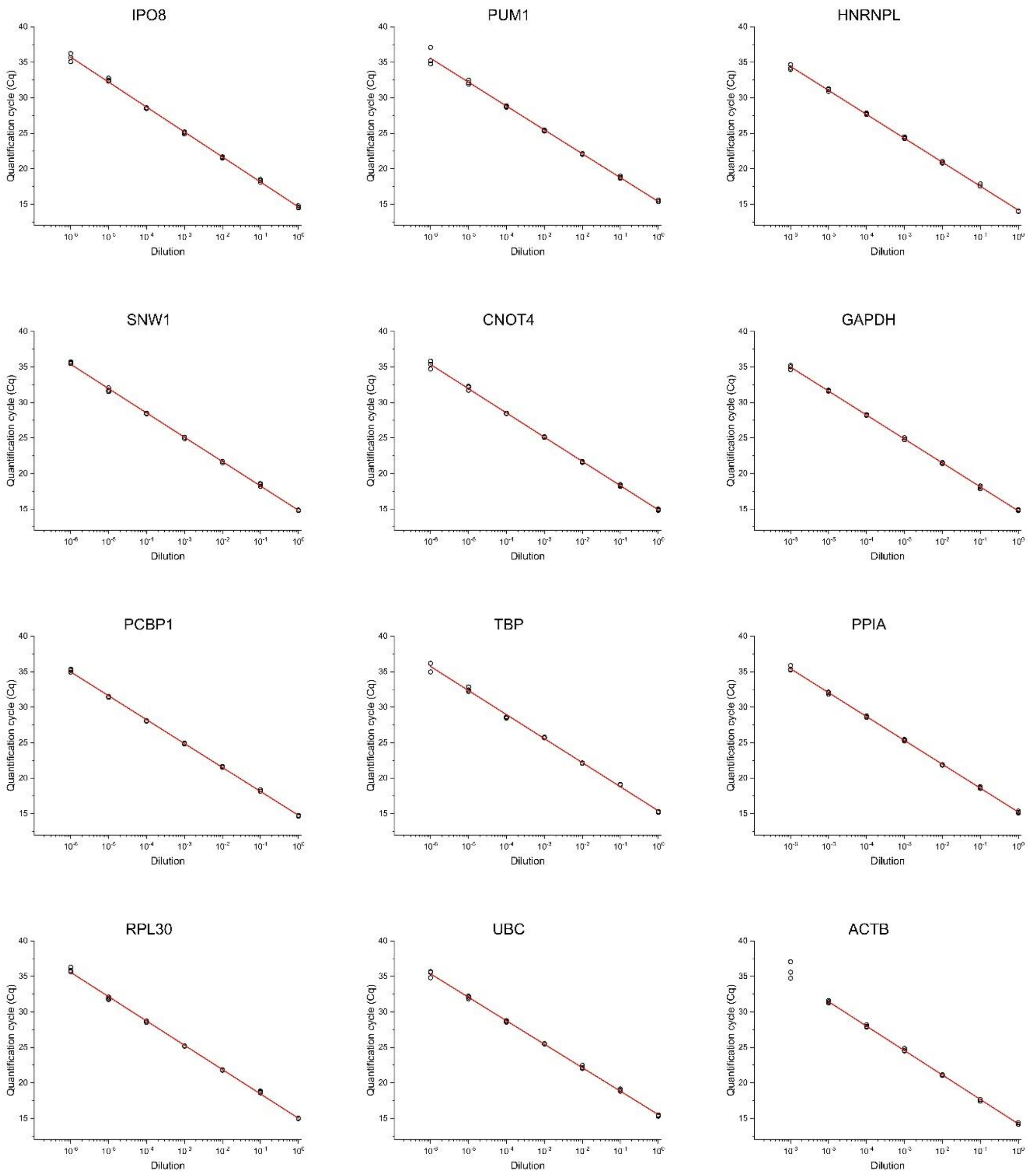

Supplementary Figure S4. Dilution curves for determination of PCR efficiency. 7 point 10-fold serial dilutions were prepared from PCR products and submitted to qPCR analysis. The Cq values for three technical replicates for each concentrartion point are marked as hollow circles. Least squares linear regression was performed to the average of the technical replicates in the indicated dilution range. The least concentrated point for ACTB was excuded from the regression. Individual graphs were created with OriginPro 2018 (OriginLab Corp.) and the figure was assembled using CorelDRAW Graphics Suite 2020 (Corel Corporation) (refer to Methods for URLs).

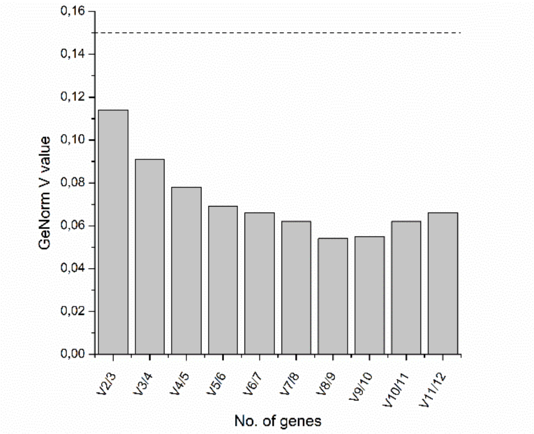

Supplementary Figure S5. Determination of the optimal number of reference targets for all cell lines investigated as indicated by the GeNorm V value. This graph was created with OriginPro 2018 software (OriginLab Corp.) (refer to Methods for URLs).

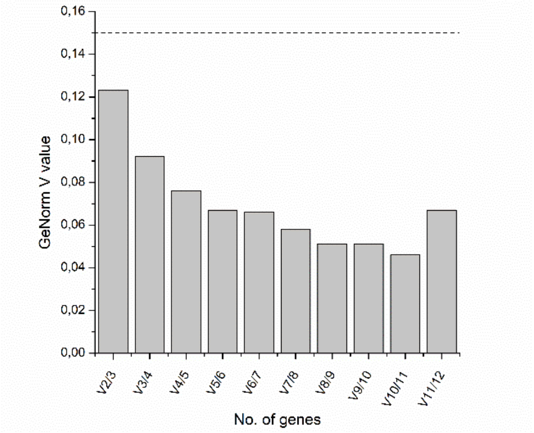

Supplementary Figure S6. Determination of the optimal number of reference targets for human cancer cell lines investigated as indicated by the GeNorm V value. This graph was created with OriginPro 2018 software (OriginLab Corp.) (refer to Methods for URLs).

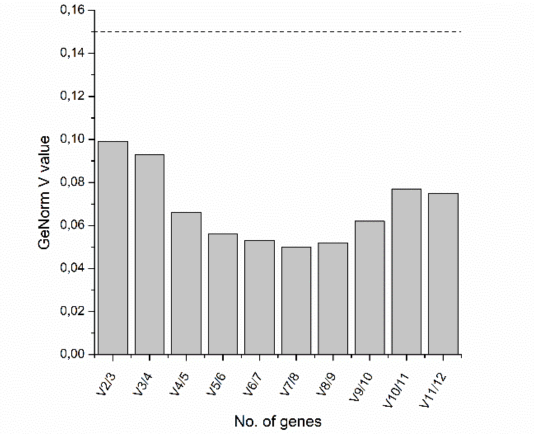

Supplementary Figure S7. Determination of the optimal number of reference targets for human normal cell lines investigated as indicated by the GeNorm V value. This graph was created with OriginPro 2018 software (OriginLab Corp.) (refer to Methods for URLs).

Supplementary Table S1. 260/280 and 260/230 ratios determined with NanoDrop for the RNA samples.

Cell line	No.	260/280	260/230	Yield (ng)
A-549	1	2.04	2.16	30330
	2	2.04	2.22	36475
	3	2.05	2.17	28695
HeLa	1	2.04	2.25	39590
	2	2.04	2.22	49350
	3	2.07	2.19	40470
HL-60	1	2.07	2.12	39585
	2	2.05	2.24	35585
	3	2.05	2.24	54335
HCT-116	1	2.06	2.18	62680
	2	2.05	2.22	54890
	3	2.06	2.24	46770
HT-29	1	2.06	2.23	33695
	2	2.08	2.23	49660
	3	2.08	2.11	59010
K-562	1	2.05	2.23	64750
	2	2.06	2.18	54880
	3	2.07	2.09	34815
MCF-7	1	2.04	2.24	50995
	2	2.05	2.07	66905
	3	2.05	2.22	40000
MDA-MB-231	1	2.07	2.04	26220
	2	2.07	2.2	37000
	3	2.06	1.95	38250
MOLT-4	1	2.07	2.19	24330
	2	2.05	2.24	33490
	3	2.03	2.23	32270
RPMI-8226	1	2.08	2.18	19800
	2	2.06	2.24	34790
	3	2.06	2.14	29840
SH-SY5Y	1	2.06	2.24	70350
	2	2.07	2.23	36790
	3	2.05	2.12	30835
U-251	1	2.05	2.24	40005
	2	2.08	2.19	17375
	3	2.05	2.16	22580
U-937	1	2.05	2.23	45820
	2	2.06	2.18	27335
	3	2.05	2.21	47865
HEK	1	2.04	2.25	54850
	2	2.05	2.24	56370
	3	2.05	2.24	63545
HFF	1	2.07	2.17	12655
	2	2.05	2.25	42570
	3	2.05	2.13	37120
HMEC	1	2.07	2.18	23990
	2	2.05	2.18	16345
	3	2.06	2.2	9875
HUES9	1	2.09	1.75	22060
	2	2.06	1.38	2195
	3	2.11	1.98	19005
HUVEC	1	2.09	1.36	3685
	2	2.07	1.67	6260
	3	2.05	1.95	6370
MRC-5	1	2.08	2.2	19000
	2	2.06	2.21	12715
	3	2.06	2.16	10370
XCL1	1	2.07	2.17	26370
	2	2.09	2.13	7130
	3	2.07	2.21	25160

HeLa serum starved	1	2.07	1.49	37720
	2	2.03	2.21	46365
	3	2.04	2.24	71100
MDA-MB-231 serum starved	1	2.07	1.53	27695
	2	2.03	2.15	33865
	3	2.02	2.06	30780
A-549 serum starved	1	2.03	2.11	31270
	2	2.04	2.16	49935
	3	2.04	2.21	78130

Supplementary Table S2. Validation of PCR efficiency using cDNA template.

	**PCR product**		**cDNA**	
**Gene symbol**	**Efficiency (%)**	**R^2**	**Efficiency (%)**	**R^2**
IPO8	92.5	0.9995	100.3	0.9956
PUM1	98.4	0.9999	98.9	0.9993
SNW1	95.8	0.9996	95.9	0.9995
GAPDH	97.8	0.9999	97.2	0.9996
PPIA	98.2	0.9999	92.3	0.9993

Supplementary Table S3. Relative expression values and the range of error based on the standard deviation for our candidate reference genes upon serum starvation and p-values as calculated by the CFX Maestro software.

Biological Group	Target	Not treated			Serum starved			p value
		Relative Quantity	RQ Lower Error Bar	RQ Upper Error Bar	Relative Quantity	RQ Lower Error Bar	RQ Upper Error Bar	
A-549	IPO8	1	0.841	1.189	0.867	0.726	1.034	0.372
	PUM1	1	0.775	1.291	1.418	1.330	1.512	0.083
	HNRNPL	1	0.830	1.205	0.499	0.483	0.515	0.003
	SNW1	1	0.856	1.168	0.627	0.588	0.670	0.009
	CNOT4	1	0.824	1.214	0.862	0.820	0.906	0.268
	GAPDH	1	0.817	1.224	0.847	0.719	0.999	0.333
	PCBP1	1	0.822	1.217	0.790	0.744	0.839	0.118
	TBP	1	0.783	1.278	0.666	0.576	0.770	0.069
	PPIA	1	0.767	1.304	1.427	1.228	1.659	0.114
	RPL30	1	0.824	1.214	1.033	0.861	1.241	0.841
	UBC	1	0.783	1.278	1.347	1.096	1.656	0.182
	ACTB	1	0.774	1.291	0.502	0.383	0.658	0.033
HeLa	IPO8	1	0.784	1.275	1.243	1.214	1.272	0.198
	PUM1	1	0.796	1.257	1.054	1.006	1.104	0.717
	HNRNPL	1	0.849	1.177	0.898	0.842	0.957	0.347
	SNW1	1	0.779	1.283	1.075	1.035	1.116	0.646
	CNOT4	1	0.744	1.345	1.008	0.928	1.096	0.965
	GAPDH	1	0.847	1.180	1.246	1.189	1.307	0.091
	PCBP1	1	0.749	1.336	1.192	1.152	1.234	0.356
	TBP	1	0.766	1.306	1.400	1.295	1.514	0.104
	PPIA	1	0.920	1.087	1.823	1.591	2.090	0.003
	RPL30	1	0.899	1.112	1.546	1.474	1.621	0.003
	UBC	1	0.890	1.124	1.402	1.277	1.539	0.017
	ACTB	1	0.828	1.207	0.806	0.721	0.901	0.164
MDA-MB-231	IPO8	1	0.847	1.181	0.737	0.654	0.830	0.061
	PUM1	1	0.896	1.116	1.237	1.168	1.309	0.041
	HNRNPL	1	0.895	1.118	0.700	0.698	0.702	0.005
	SNW1	1	0.858	1.165	0.941	0.792	1.119	0.674
	CNOT4	1	0.925	1.081	1.000	0.984	1.017	0.997
	GAPDH	1	0.880	1.136	1.311	1.249	1.377	0.026
	PCBP1	1	0.909	1.100	0.951	0.818	1.107	0.654
	TBP	1	0.867	1.153	0.644	0.560	0.740	0.019
	PPIA	1	0.830	1.205	1.311	1.177	1.460	0.096
	RPL30	1	0.940	1.064	0.854	0.801	0.912	0.039
	UBC	1	0.885	1.130	1.778	1.461	2.164	0.013
	ACTB	1	0.834	1.199	0.775	0.660	0.910	0.142
